# Divergence of C4A and C4B in first-episode psychosis: Insights from CSF and plasma immune profiling

**DOI:** 10.1038/s41398-026-04037-y

**Published:** 2026-04-18

**Authors:** Shokouh Arjmand, Mridul Chaudhary, Samudyata, Fredrik Piehl, Aurimantas Pelanis, Göran Engberg, Simon Cervenka, Mikael Landén, Sophie Erhardt, Carl M. Sellgren

**Affiliations:** 1 Department of Physiology and Pharmacology, Karolinska Institutet, Stockholm, Sweden; 2 Translational Neuropsychiatry Unit, Department of Clinical Medicine, Aarhus University, Aarhus, Denmark; 3 Neuroimmunology Unit, Department of Clinical Neuroscience, Karolinska Institutet, and Neuro Division, Karolinska University Hospital, Stockholm, Sweden; 4 Department of Anesthesiology, Sahlgrenska University Hospital, Gothenburg, Sweden; 5 Institute of Sport Science and Innovations, Lithuanian Sports University, Kaunas, Lithuania; 6 Centre for Psychiatry Research, Department of Clinical Neuroscience, Karolinska Institutet & Stockholm Health Care Services, Region Stockholm, Stockholm, Sweden; 7 Department of Medical Sciences, Psychosis Research and Preventive Psychiatry, Uppsala University, Uppsala, Sweden; 8 Institute of Neuroscience and Physiology, University of Gothenburg, Gothenburg, Sweden; 9 Department of Medical Epidemiology and Biostatistics, Karolinska Institutet, Stockholm, Sweden

**Keywords:** Schizophrenia, Molecular neuroscience

## Abstract

The complement genes *C4A* and *C4B* share high sequence similarity yet differ in biological function and disease relevance. C4A, in contrast to C4B, is implicated in synaptic pruning and increased risk for schizophrenia, however beyond this their distinct roles within the human brain remain poorly understood. We analyzed cerebrospinal fluid (CSF) levels of C4A, C4B, C1Q, along with 48 inflammation-related proteins, measured in both CSF and plasma, in 90 healthy controls and 113 patients with first-episode psychosis (FEP). In controls, C4A and C4B were positively associated with C1Q (z = 0.41, p < 0.001, and z = 0.48, p < 0.001, respectively), whereas in FEP, the CSF C1Q–C4A association was abolished (z = 0.09, p = 0.40). Across inflammatory markers, C4A levels in controls showed predominantly negative correlations in CSF, while C4B and C1Q exhibited mostly positive correlations. Using permutation tests on directional mean differences, we observed a robust positive directional shift for C4A in FEP (z = 3.81, *p* < 0.0001), while C4B showed a non-significant negative shift (z = −2.64, *p* > 0.9). The overall C4A directional shift was largely preserved in plasma, though the structure of complement–protein interactions differed markedly between the biological compartments, CSF and plasma. Together, these findings identify distinct and compartment-specific patterns of immune network interactions for C4A and C4B and suggest that C4A–inflammatory protein relationships are selectively altered in FEP.

## Introduction

The complement system is a well-recognized regulator of the innate and adaptive immunity with more than 40 soluble and membrane-associated components [1]. Although typically associated with immediate immune response, mounting evidence suggests that the complement system and its individual components also play alternative roles in the brain [1]. Among these, one of the most extensively studied is the involvement of various complement components in removing redundant synapses during brain development [2, 3]. Genetic ablation of the key components of the classical pathway (CP) has been demonstrated to reduce the internalization of synaptic structures by microglia, implicating signaling through the CP as a mechanism repurposed in the brain to facilitate synaptic pruning [3–6].

In patients with schizophrenia, a decrease in synapse density can be observed [7, 8]. Patient-derived cellular models further suggest an excessive internalization of synaptic structures by microglia [9]. Moreover, genome-wide association studies have identified a risk locus within the extended major histocompatibility complex region, which harbors the two genes encoding C4A and C4B [4]. Specifically, the genetic risk associated with this locus has largely been attributed to increased copy numbers (CNs) of *C4A*, while repeats of *C4B* do not increase schizophrenia risk [4]. Recently, we also detected elevated protein levels of C4A, but not C4B, in cerebrospinal fluid (CSF) obtained from patients with first-episode psychosis (FEP) [10], while in an ensuing study of the same overlapping sample, C1q levels were lower [11]. As C4A has also been shown to bind synapses with higher affinity than C4B [5, 12], this suggests that C4A and C4B display functional specialization in the human brain, with a possible relevance for synapse elimination in schizophrenia. However, the functional implications of differential C4A and C4B levels in the brain remain largely unexplored, and an elevation in C4A levels may also reflect a broader immune dysregulation beyond the CP.

In this study, we therefore asked two main questions: 1) how do C4A and C4B protein levels in the human central nervous system relate to other immune-related proteins within and beyond CP, and 2) are these associations altered in FEP? To address these questions, we analyzed data from a large cohort of individuals with FEP (n = 113), and HC (n = 90) recruited through the Karolinska Schizophrenia Project (KaSP) and the Gothenburg Research Initiative on Psychosis (GRIP). Among the complement proteins, we focused on CSF C1Q levels, the initiator of the CP, which we recently found to be reduced in FEP. Additionally, non-complement immune-related proteins were measured in plasma and CSF using the OLINK Proteomics- Inflammation Panel [13], previously shown to primarily reflect altered plasma levels in FEP patients [14].

## Methods

### Participants

This research was approved by the Stockholm Regional Ethics Committee (Dnr 2010/879–31/1, 2005/554-31/3, and 2013/43-32). All participants provided a written informed consent prior to the initiation of the study, adhering to the Declaration of Helsinki. The study included both HCs and patients with FEP, recruited as part of the KaSP (n = 98) and the GRIP (n = 105) [10]. In total, CSF from 90 HCs and 113 patients with FEP was used in this study and demographic and clinical characteristics of the participants are presented in Table 1. Detailed clinical characteristics of the participants, stratified by cohort, are provided in Table S1.

**Table 1:** Demographic and clinical characteristics of the participants.

		Valid	p values	Mean	Std. Deviation	95% Confidence Interval Mean	
						Lower	Upper
Sex (F/M)	HCs	90 (42/48)	0.136	–	–	–	–
	FEPs	113 (41/72)		–	–	–	–
Age	HCs	90	0.006	37.66	14.22	34.68	40.63
	FEPs	113		31.94	10.03	30.07	33.80
BMI (kg/m^2^)	HCs	88	0.920	24.68	3.58	23.92	25.44
	FEPs	111		24.69	4.25	23.89	25.49
Nicotine use	HCs	88 (15/88)	0.020	–	–	–	–
	FEPs	87 (29/87)		–	–	–	–
PANSS Positive	HCs	NA					
	FEPs	90		17.61	6.12	16.33	18.89
PANSS Negative	HCs	NA					
	FEPs	90		16.50	6.59	15.12	17.88
PANSS General	HCs	NA					
	FEPs	90		35.99	10.48	33.79	38.18
PANSS Total	HCs	NA					
	FEPs	90		70.10	18.99	66.12	74.08
GAF-S	HCs	52		84.58	7.71	82.43	86.72
	FEPs	67		33.63	10.34	31.10	36.15
GAF-F	HCs	52		85.11	7.17	83.12	87.11
	FEPs	67		41.82	13.07	38.63	45.01
CGI	HCs	NA					
	FEPs	72		4.53	1.20	4.25	4.81
DUP (Months)	HCs	NA					
	FEPs	58		11.26	6.56	15.95	17.85

*PANSS*, Positive and Negative Syndrome Scale; *GAF*, Global Assessment of Functioning (Symptoms and Function); *CGI*, Clinical Global Impression; *DUP*, Duration of Untreated Psychosis; *NA*, Not Applicable.

#### Patients

A total of 113 patients, 68 from the KaSP cohort and 45 from the GRIP cohort (41 females and 72 males), with FEP were included in the study. In the KaSP study, a diagnosis was made based on a structured clinical interview using the DSM-IV criteria and discrepancies were discussed to reach a consensus diagnosis. In the GRIP cohort, patients were diagnosed through structured clinical interviews and review of medical records. Final diagnoses were confirmed in consensus case conferences involving at least two board-certified psychiatrists. Patients with neurological disorders, severe general medical conditions, a history of substance abuse except nicotine-based products, or co-existing neurodevelopmental disorders were excluded. Substance use was evaluated through urine tests, and MRI scans were used to rule out significant brain abnormalities. 36 patients were antipsychotic-naïve, while 77 of them were receiving antipsychotics at the time of sample collection. Clinical characteristics of the participants were assessed using several scales, including the Global Assessment of Functioning (GAF), Positive and Negative Syndrome Scale (PANSS), Clinical Global Impression (CGI), Alcohol Use Disorders Identification Test, and Drug Use Disorders Identification Test. Moreover, follow-up of patients with FEP indicated that approximately 60% were later diagnosed with schizophrenia. Additional cohort characteristics have been described previously [10].

#### Controls

A total of 90 healthy participants, 37 from the KaSP cohort and 53 from the GRIP cohort (42 females and 48 males) were recruited through advertisements for the study or selected by Statistics Sweden based on matching. Various assessments were conducted, including a physical examination, MRI, and blood and urine tests. The Mini International Neuropsychiatric Interview was used to screen for any prior psychiatric conditions. Additional exclusion criteria included any history of illegal substance use and having first-degree relatives with psychotic disorders. At the time of the study, none of the healthy participants were on medication or had a history of substance abuse, as confirmed by the Alcohol and Drug Use Disorders Identification Tests. None of the participants had first-degree relatives with psychiatric conditions. In KaSP, controls were recruited through advertisement with the aim of achieving sex- and age-matching. In GRIP, controls were selected by Statistics Sweden to largely reflect mean age and sex distribution in the larger St. Göran Projekt [15] (in which GRIP is a sub study).

### Blood sampling

Venous blood samples were collected in 10-ml tubes containing EDTA (BD Vacutainer; BD Hemograd, K2 EDTA). The samples were centrifuged at 2900 rpm for 15 min within one hour of collection and stored at −80 °C until further analysis. Participants were instructed to refrain from physical activity for at least eight hours prior to sample collection, and blood samples were drawn in the morning.

### CSF collection

Study participants fasted overnight, and an experienced neurologist or anesthetist (mainly A.P. and F.P.) performed the lumbar puncture the following morning using a disposable atraumatic 22 G spinal needle inserted at the L4–5 interspace, collecting 15–18 mL of CSF. All collected CSF samples were clear and colorless upon visual inspection. Within 1 h after the lumbar puncture, the CSF was centrifuged at 3500 rpm for 10 min. The resulting cell-free CSF was then aliquoted and stored at –80 °C until analysis. Each CSF sample was analyzed individually.

### Targeted liquid chromatography-mass spectrometry (LC-MS) (C4A, C4B, and C1QA)

**Fig. 1:**
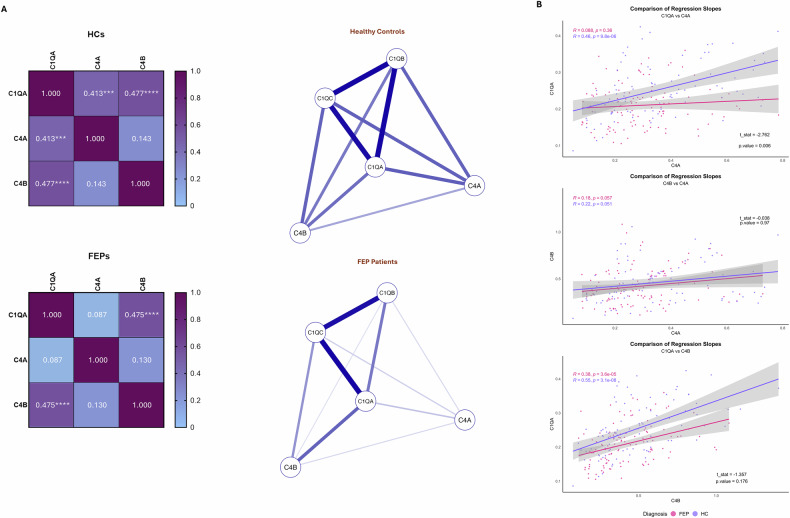
Correlations of CSF complement factors with each other. Panels **A** and **B**: Correlation matrices and network analyses of the associations between CSF protein levels of C4A, C4B, and C1Q in healthy controls (HCs) and first-episode psychosis (FEP) patients, adjusted for age, sex, and BMI. Differences in slopes between diagnostic groups (FEP vs HC) were calculated to illustrate differences in association strength between FEP and HC. Regression lines and group-wise data points and the statistics are depicted in panel C. C4A significantly loses its association with C1Q in FEP. Moreover, C4A and C4B are not correlated in either HCs or in FEP.

Sample preparation was carried out using the Agilent AssayMAP Bravo Platform, with detailed procedures described in previous publications [11]. In brief, 50 µg of total protein from each CSF sample was used for targeted LC-MS analysis of a selection of unique tryptic peptides of C4A, C4B, and C1QA. Given the high inter-correlation of C1Q subtypes in HCs, we selected C1QA for further analyses (Fig. 1 and S1). All raw data generated on the Fusion MS were imported to Skyline v4.1 (MacCoss Lab Software, USA) for analysis. Peak integration was performed automatically by the software and manually reviewed to ensure accurate peak detection.

### OLINK analyses (CSF and plasma)

Proteins included in the OLINK Target Inflammation panel, based on multiplex proximity extension assay (PEA) technology, were analyzed by OLINK Bioscience (Uppsala, Sweden), as previously described in detail [14]. Briefly, 92 inflammatory markers were measured in CSF and plasma using a microtiter plate containing 96 pairs of DNA-labeled antibody probes per well. The PEA technology relies on pairs of oligonucleotide-conjugated antibodies that bind to specific target proteins. Upon binding, the antibody probes are brought into close proximity, enabling DNA polymerization. The resulting DNA sequence is amplified and quantified using real-time PCR, serving as a surrogate for the corresponding protein concentration. PCR results were normalized for readout variability between the plates using interplate controls (IPC). Final data were reported as Normalized Protein eXpression (NPX), an arbitrary unit based on log2 transformation, reflecting relative protein levels across samples.

Forty eight of the 92 proteins were detectable in CSF within the cohort. Consequently, we restricted our plasma analyses to these matched proteins to enable a direct comparison across biological compartments.

### Molecular analyses of *C4* structural elements (Droplet Digital PCR)

Using droplet digital PCR **(**ddPCR), CNs of *C4* structural elements (*C4A*, *C4B*, *C4-HERV* CNs) in the GRIP cohort were measured from genomic DNA isolated from blood samples. *C4A-HERV* + , *C4A-HERV* − , *C4B-HERV* + , and *C4BHERV* − *CN*s were determined based on imputations. A detailed protocol for imputations can be found in an earlier publication [10] and on Github: https://github.com/freeseek/imputec4.

### Molecular analyses of *C4* structural elements (imputation from whole genome sequencing)

CNs of *C4* structural elements (*C4A-HERV* + , *C4A-HERV* − , *C4B-HERV* + , and *C4B-HERV* − CNs) in the KaSP cohort were imputed from MHC genotypes computed from whole genome sequencing (WGS) data as described earlier. HapMap3 CEU reference haplotype panel, from Sekar et al. [4], was used to impute pre-annotated *C4* haplogroups, using Beagle (version 3.3), with subsets of SNPs in the extended MHC locus (chr6: 25–34 Mb).

### Statistical analysis

Statistical analyses were conducted using R for Mac OS (version 4.5.2), JASP version 0.19.1 (Apple Silicon) and GraphPad Prism version 10.4.2. Pearson’s correlation was used to assess relationships between normally distributed continuous variables, while Spearman’s correlation was applied for non-normally distributed variables. Normality was assessed using the Shapiro-Wilk test. Missing values were handled using pairwise deletion to maximize the available data for each correlation (Table S2).

To correct for multiple comparisons, we controlled the false discovery rate (FDR) using the two-stage linear step-up procedure of Benjamini, Krieger, and Yekutieli, applying a *q*-value threshold of 10%. The Brown-Forsythe test was used to evaluate the equality of variances. Depending on the data distribution and variance, appropriate statistical tests were applied, including Student’s t-test, Welch’s t-test, or the Mann-Whitney U test. A two-tailed p-value of less than 0.05 was considered statistically significant with asterisks denoting p values (*p < 0.05, **p < 0.01, ***p < 0.001, ****p < 0.0001). The network structure was estimated using zero-order Spearman correlations, without regularization, to capture direct relationships between variables. To evaluate the stability of the estimated edges, nonparametric bootstrapping with 10,000 resamples was performed to construct confidence intervals around the correlation-based edge weights.

All statistical analyses included age, sex, antipsychotic medication use, and BMI as covariates. Notably nicotine use was excluded as a covariate based on a preliminary analysis (Fig. S2).

Linear regression models were fitted to compare complement relationships between patients with FEP and HCs, modeling one complement protein as a function of another. Group-specific slopes were extracted and compared using a two-sided Welch t-test. Results were visualized using group-wise scatter plots with fitted regression lines.

For each protein in the inflammation panel, partial Spearman correlations were calculated between CSF and plasma inflammatory protein levels (OLINK) and CSF complement factors, adjusting for age, BMI, sex, and antipsychotic medication use. Analyses were performed separately in patients with FEP and HCs. Differences in associations between groups were quantified as the difference between group-specific partial correlation coefficients (cor_partial_ (FEP) - cor_partial_ (HC)). Statistical significance of these differences was assessed using Fisher’s z-transformation with group-specific sample sizes. The resulting p-values reflect evidence against the null hypothesis of equal correlations between patients and controls for each protein–complement pair. These results were visualized using hub-and-spoke plots.

To compare overall association patterns between CSF complement factors and the full set of inflammatory proteins across diagnostic groups (FEP vs HC), differences in correlation distributions were evaluated using Wilcoxon rank-sum tests. Global shifts in correlations across all proteins were further assessed using permutation-based mean-difference tests. Directional tests examined whether correlations were consistently higher or lower in FEP compared with HCs, whereas non-directional tests assessed overall changes in correlation magnitude. Statistical significance was determined using empirical p-values derived from permutation-based null distributions.

Principal component analysis (PCA) was performed on scaled inflammatory protein levels to reduce dimensionality and summarize shared variation across markers. The proportion of variance explained by each component was examined. The first four principal components were retained for downstream analyses. Protein loadings were used to identify major contributors to each component. To assess clinical relevance, scores of the selected principal components were correlated with PANSS positive, negative and general.

## Results

### Associations between complement proteins in CSF

In HCs (n = 90), CSF levels of both C4A and C4B correlated robustly with CSF C1QA levels (z = 0.41, p < 0.001 for C4A, and z = 0.48, p < 0.001 for C4B; Fig. 1A). However, no significant correlation was observed between C4A and C4B levels (z = 0.14; p = 0.22; Fig. 1A).

In patients with FEP (n = 113), the CSF levels of C4B and C1QA were strongly correlated (z = 0.475, p < 0.001), mirroring the pattern observed in HCs (Fig. 1A). In contrast, the correlation observed between C4A and C1QA levels was absent in FEP patients (z = 0.09, p = 0.40; Fig. 1B). As in the HC group, no significant correlation was observed between C4A and C4B levels in patients with FEP (z = 0.13, p = 0.19; Fig. 1A). Comparing the correlations in FEP patients versus HCs, only the correlation between C4A and C1Q was significantly different (Welch’s t = −2.762, p = 0.006; Fig. 1B). After adjusting for age, sex, BMI, and use of antipsychotic medication, the absence of a C4A–C1Q association in FEP patients persisted (Fig. S3). Further, restricting the analyses to antipsychotic-naïve FEP patients, with additional adjustment for age, sex, and BMI, yielded a highly similar pattern (Fig. S3).

### C4A/C4B levels and inflammation markers in CSF – profile-level comparisons

**Fig. 2:**
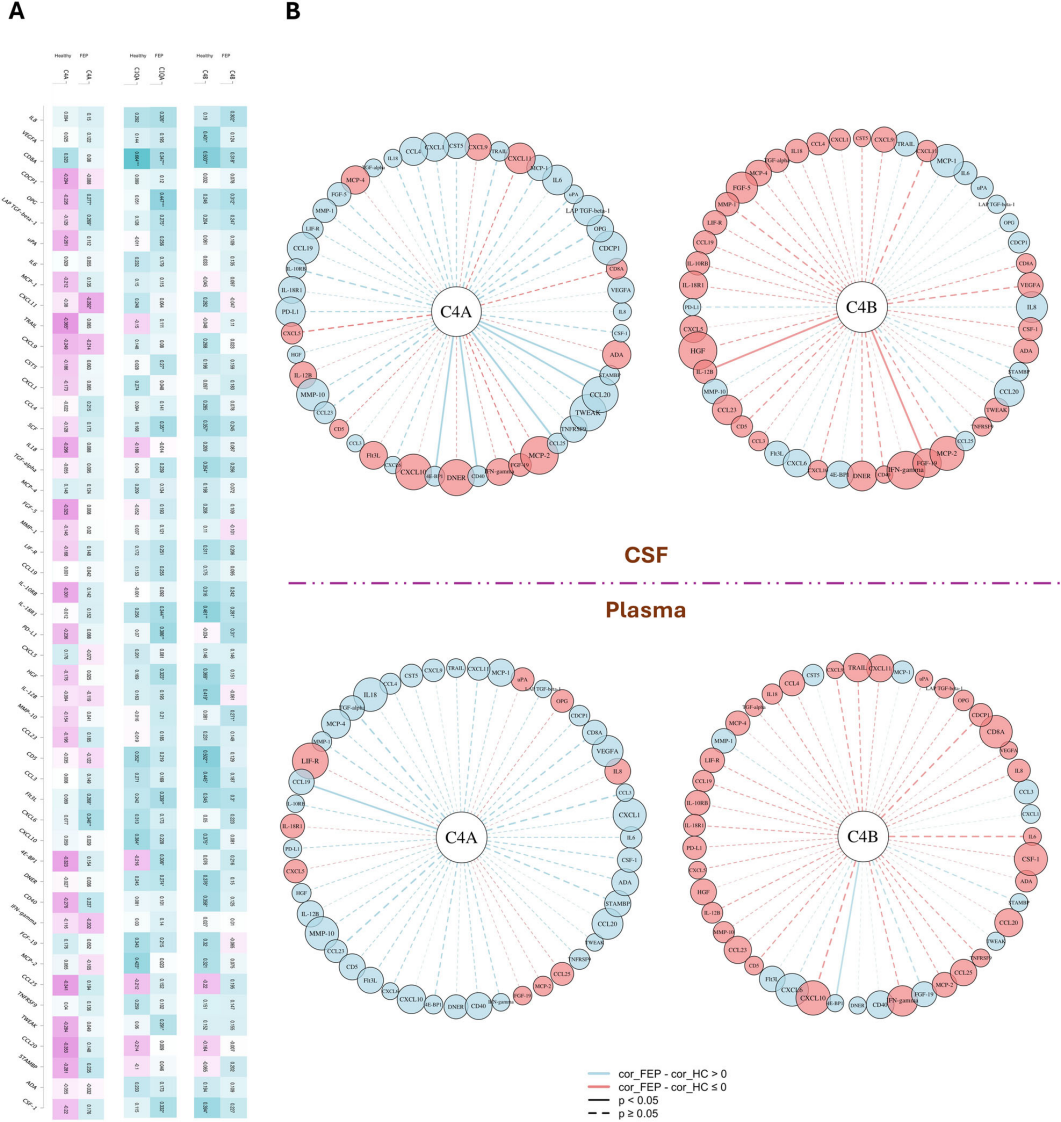
Complement-centered inflammatory association patterns across biological compartments in patients with first-episode psychosis (FEP) with reference to healthy controls (HCs). Panel **A**: Heatmaps displaying partial correlations between CSF levels of C4A, C4B, and C1QA and 48 inflammatory proteins detected in CSF, adjusted for BMI, in HCs and patients with FEP, illustrating a distinct set of associations for C4A in HCs that is altered in FEP. Positive and negative correlations are represented by cool and warm colors, respectively, with the strength of the correlation indicated by the intensity of the color. Panel **B**: The hub-and-spoke plots depict each complement (hub) at the center connected to all measured inflammatory proteins (spokes). Edge color represents the direction of the correlation difference between patients and controls (light blue indicates a more positive association in FEP patients, while light coral points to a more positive correlation in HCs). Edge thickness is proportional to the absolute magnitude of this difference. Surrounding protein nodes (spokes) are colored to match the direction of the edges and their size is scaled proportionally to the correlation difference, analogous to edge thickness. Solid edges denote statistically significant differences, while dashed edges indicate non-significant differences. C4A and C4B show distinct complement-specific inflammatory association profiles across CSF and plasma. Among these, C4A exhibits the most pronounced and consistent divergence in FEP patients relative to HCs (z = 3.81, p < 0.0001), with a greater number of inflammatory proteins showing more positive effects in FEP compared with the largely preserved and unaffected association patterns observed for C4B in FEP relative to HCs (z = −2.64, p > 0.9). Although the overall direction shifts are broadly consistent across compartments, the organization of individual complement–protein associations differ substantially between CSF and plasma.

In HCs (n = 35), C4A displayed a pattern of predominantly negative correlations with the 48 proteins in the OLINK Inflammation panel, whereas C4B largely showed positive correlations (Fig. 2A). Contrary to HCs, in FEP patients (n = 63), correlations between C4A and this set of inflammatory proteins shifted to mostly positive values, resembling those of C4B (Fig. 2A). For comparison, correlations between C1Q and the same inflammatory proteins mirrored C4B patterns in both HCs and FEP patients (Fig. 2A). To also evaluate to what extent variations in CNs of *C4A* and *C4B* influenced the correlations, we performed correlation analyses in a subset with *C4A* and *C4B* structural elements analyzed by ddPCR (22 HCs and 45 FEP patients). The general correlation patterns remained largely similar both for C4A and C4B in HCs as well as in FEP, with a tendency towards more pronounced negative correlations for C4A was observed in HCs, but with a positive shift in FEP as in the unadjusted analyses (Fig. S4).

To test the statistical significance of these disease-related profile-level changes for C4A and C4B in the whole sample, we applied permutation-based mean-difference tests. The absolute mean difference in Spearman correlations revealed robust increases in association strength for both C4A and C4B in FEP versus HCs (p < 0.0001 for each).

To assess whether these global differences reflected systematic directional shifts (rather than magnitude alone), we employed permutation tests on directional mean differences (FEP vs. HCs). C4A showed a pronounced positive shift across the inflammatory profile (z = 3.81, p < 0.0001), whereas C4B exhibited a modest negative shift, albeit nonsignificant (z = −2.64, p > 0.9).

### C4A/C4B levels and inflammation markers in CSF – individual protein correlations

To further delineate which individual inflammatory proteins contributed to the global complement–inflammation profile differences, we examined protein-specific correlation differences (Δcor _FEP-HC_) adjusted for age, sex, BMI, and antipsychotic medication use (Fig. 2B). Consistent with permutation tests, C4A displayed pronounced directional asymmetry, with more inflammatory proteins showing positive (Δcor _FEP-HC_); significant associations emerged with STAMBP, CCL20, CD40, 4E-BP1, and CCL25 (Fig. 2B). In contrast, C4B exhibited predominantly negative (Δcor _FEP-HC_), indicating attenuated associations in FEP, with significant correlations only for IL-12B and FGF-19 (Fig. 2B).

### C4A/C4B levels and plasma inflammation markers

Given our previous case-control differential analyses of the OLINK-inflammation panel in CSF and plasma, displaying foremost elevated protein levels in plasma [14], we also analyzed plasma OLINK inflammatory proteins against CSF C4A and C4B levels.

Similar to CSF OLINK analyses, we applied permutation-based absolute and directional mean difference tests (profile-level), followed by analyzing protein-specific (Δcor _FEP-HC_) (CSF C4A or CSF C4B × disease status) from individual linear models adjusted for age, sex, BMI, and antipsychotic medication use. Mirroring the CSF inflammatory panel results, the absolute mean difference in Spearman partial correlations revealed robust increases in association strength for both C4A and C4B in FEP (p < 0.0001 for both), with a large and significant positive directional shifts for C4A (z = 5.13, p < 0.0001) and a more modest and non-significant directional shift for C4B (z = −3.08, p > 0.9).

Examining protein-specific (Δcor _FEP-HC_) (disease status) for C4A (Fig. 2B), the significant positive difference observed in CSF with STAMBP, CCL20, CD40, and 4E-BP1 remained positive in plasma but lost statistical significance, while the difference with CCL25 shifted from positive to negative, though none-significant (Fig. 2B). In plasma, CCL19 was the only protein exhibiting a significant (Δcor _FEP-HC_) with C4A. Regarding C4B, the significant positive (Δcor _FEP-HC_) observed in CSF with IL-12B remained positive but non-significant in plasma, while the FGF-19 correlation shifted to a non-significant positive direction (Fig. 2B). The only plasma protein maintaining a significant (Δcor _FEP-HC_) with CSF C4B was 4E-BP1 (Fig. 2B). Comparison of association patterns across the full plasma protein set is shown in Fig S5.

To interpret these findings in the context of the plasma versus CSF compartments, we conducted correlation analyses to assess the correspondence between CSF and plasma levels of the selected proteins from the OLINK inflammation panel. Overall, correlation coefficient largely clustered near zero, indicating a generally weak association across the panel, with only a small subset, comprising 9 proteins in FEP patients and 3 proteins in HCs, reaching significance (Table 2).

**Table 2:** CSF and plasma represent biologically distinct immune compartments.

Inflammatory Proteins	Controls		FEP	
	Effect size	p value	Effect size	p value
IL8	-0.332	0.055	0.060	0.657
VEGFA	-0.129	0.468	-0.082	0.530
CD8A	-0.379	0.027	0.047	0.718
CDCP1	-0.038	0.832	0.073	0.575
OPG	-0.018	0.921	0.069	0.597
LAP TGF-beta-1	-0.213	0.226	0.064	0.726
uPA	0.167	0.346	-0.030	0.816
IL6	-0.155	0.380	0.149	0.251
MCP-1	-0.187	0.288	0.162	0.211
CXCL11	-0.018	0.920	0.064	0.625
TRAIL	0.097	0.584	0.241	0.061
CXCL9	0.286	0.100	0.571	0.000
CST5	-0.158	0.374	0.079	0.547
CXCL1	-0.343	0.047	0.032	0.809
CCL4	0.235	0.182	0.114	0.383
SCF	-0.324	0.062	0.027	0.839
IL18	0.187	0.291	0.183	0.158
TGF-alpha	-0.181	0.305	-0.138	0.288
MCP-4	0.060	0.735	0.191	0.140
MMP-1	-0.067	0.705	0.328	0.010
LIF-R	0.025	0.887	-0.069	0.589
CCL19	-0.138	0.436	0.153	0.238
IL-10RB	-0.201	0.255	0.191	0.140
IL-18R1	-0.006	0.975	0.312	0.014
PD-L1	-0.169	0.339	-0.009	0.947
CXCL5	-0.171	0.185	0.013	0.921
HGF	-0.150	0.397	0.163	0.209
IL-12B	0.100	0.573	0.318	0.012
MMP-10	0.179	0.310	0.260	0.043
CCL23	0.196	0.267	0.153	0.240
CD5	0.029	0.869	0.126	0.332
CCL3	-0.018	0.921	0.198	0.125
Flt3L	-0.102	0.567	0.090	0.490
CXCL6	-0.441	0.009	0.161	0.216
CXCL10	0.137	0.440	0.132	0.309
4E-BP1	0.106	0.551	-0.144	0.269
DNER	-0.066	0.710	-0.135	0.298
CD40	-0.225	0.200	0.100	0.443
IFN-gamma	0.263	0.133	0.320	0.012
FGF-19	-0.163	0.357	0.062	0.634
MCP-2	-0.041	0.816	0.243	0.059
CCL25	-0.164	0.355	-0.299	0.019
TNFRSF9	-0.173	0.328	0.200	0.122
TWEAK	-0.006	0.975	0.098	0.453
CCL20	0.092	0.604	0.534	0.000
STAMBP	-0.085	0.631	-0.226	0.080
ADA	-0.072	0.686	0.255	0.047
CSF-1	-0.085	0.632	-0.079	0.546

Correlation analyses were performed for 48 proteins measured in paired CSF and plasma samples in FEP patients and healthy controls. Pink colored are proteins exhibiting statistically significant correlations between CSF and plasma concentrations within each group (p < 0.05). The majority of proteins did not show significant correlations, indicating largely independent regulation of protein levels across biological compartments. Proteins significantly correlated in patients and controls were non-overlapping, highlighting disease-dependent and selective peripheral–central coupling rather than global concordance between CSF and plasma.

### Combining complement proteins and inflammatory proteins in relation to symptom severity

**Fig. 3:**
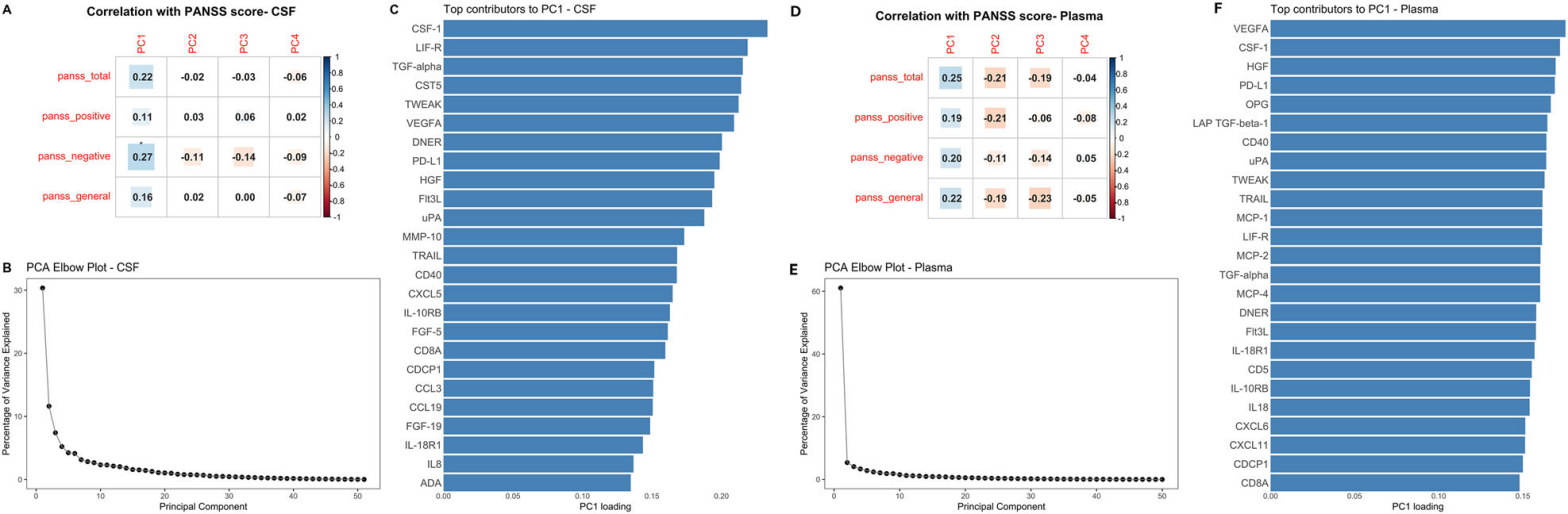
Principal component analysis of complement and inflammatory proteins in CSF and plasma. **A** Correlations between the first four principal components (PC1–PC4) and clinical PANSS scores. **B** Elbow plot showing the proportion of variance explained by the leading principal components. **C** Bar plot of the top protein loadings contributing to PC1, highlighting the proteins that most strongly drive the primary axis of variation. **D** Correlations between the first four principal components (PC1–PC4) and clinical PANSS scores. **E** Elbow plot showing the proportion of variance explained by the leading principal components. **F** Bar plot of the top protein loadings contributing to PC1, highlighting the proteins that most strongly drive the primary axis of variation.

To investigate whether combined CSF measurements of complement proteins (C4A, C4B, and C1Q) and the set of inflammatory proteins were associated to symptom severity in FEP, we first performed a PCA in which the first principal component (PC1) accounted for 30.3% of the total variance, while PC2 explained an additional 11.6% (Fig. 3B). PC1 showed positive correlations with all PANSS domains, reaching statistical significance for PANSS Negative symptoms (r = 0.27, p = 0.03; Fig. 3A). However, this association did not survive correction for multiple comparisons, appearing only as a trend (p = 0.07). Examination of PC1 loadings indicated that CSF-1, LIF-R, TGF-α, CST5, and TWEAK were the strongest contributors, suggesting that variation in this subset of proteins primarily underlies the observed association between CSF PC1 and negative symptom severity (Fig. 3C).

Combining CSF complement protein measurements with the inflammatory proteins measured in plasma, PC1 captured a substantially larger proportion of variance, explaining 61% of the total variability across proteins (Fig. 3E). PC1 was positively correlated with all PANSS domains but did not exhibit any significant association with PANSS domains (p = 0.14 for all domains; Fig. 3D). The top contributors to PC1 were VEGFA, CSF-1, HGF, PD-L1, and OPG (Fig. 3F).

## Discussion

By studying CSF levels of C4A, C4B, and C1Q alongside inflammatory proteins in CSF and plasma from HCs and FEP patients, we discovered that: (1) CSF C4A–C1Q correlation is selectively absent in FEP; (2) unlike C4B andC1Q, C4A shows predominantly negative associations with most inflammation-related proteins; (3) C4A correlations exhibit a robust positive directional shift in FEP, while C4B correlations show a non-significant negative shift (in both compartments); and (4) despite similar profile-level directional patterns, individual protein–protein correlations underlying these shifts differ between CSF and plasma.

The selective loss of positive C4A–C1Q protein correlation in FEP (1) mirrors prior postmortem findings on mRNA expression in brain tissues from schizophrenia patients [11]. Notably, both C1Q mRNA (schizophrenia) and protein (FEP and first-episode schizophrenia) levels are reduced, indicating that, unlike genetically driven *C4A* upregulation, C1Q undergoes negative feedback, likely as a damage-limiting response to consumption. This raises the intriguing possibility that C4A-driven synaptic opsonization can become uncoupled from C1q under disease conditions. Consistent with this notion, C4 activation can occur via the lectin pathway, where MASPs replace C1s (downstream to C1Q) [16]. Moreover, C1q-independent synapse elimination (e.g., post-viral infection) has been reported [1, 17]. Future studies should therefore test whether complement-dependent mechanisms persist in such contexts.

The observation that C4A, unlike C4B, largely shows weak negative correlations with “pro-inflammatory” proteins under normal conditions (2) strengthens the idea that C4A is more coupled to functions beyond classical immune signaling. This aligns with experimental data showing that C4A binds synapses more effectively than C4B [4, 9].

In FEP, C4A correlations exhibit a robust positive directional shift (3), including significant associations with STAMBP, CCL20, CD40, 4E-BP1, and CCL25. The cross-sectional nature of this study precludes determining causality, specifically, whether elevated C4A drives these immune proteins’ profile or if FEP-related immune activation induces *C4A*. Nevertheless, our prior work demonstrated that IL-1β and IL-6 selectively upregulate neuronal *C4A* mRNA, and we observed a significant correlation between CSF C4A and IL-1β in FEP [10]. In the current study, the IL-6–C4A correlation was weak, albeit enhanced in the FEP group. Instead, C4A correlated positively with STAMBP, CCL20, CD40, 4E-BP1, and CCL25. Given that IL-1β can act as a central indirect or direct upstream driver of these proteins, our results are consistent with a model in which IL-1β potentially drives *C4A* expression in FEP relative to gene copy number, potentially further enhancing excessive synaptic loss.

Notably, the absence of a directional shift for C4B, despite similarly large absolute changes in correlation strength, underscores that directional bias, rather than overall magnitude, drives the divergence of C4A from C4B.

Despite parallel profile-level directional patterns, individual complement–inflammation correlations differed between CSF and plasma (4). These findings indicate largely preserved compartment-specific regulation in both HCs and patients with FEP. Even under disease conditions, where systemic immune activation might be expected to influence central immune profiles, we found that fewer than one-fifth of the 48 inflammatory proteins showed significant CSF–plasma coupling. Similar central–peripheral immune dissociations have been reported previously [18]. Specifically, Gallego et al. have reported poor CSF–plasma C4 correlation in schizophrenia [19]. These observations underscore that systemic measurements may capture only a fraction of the biological variability relevant to brain processes and highlight the necessity of a cautious interpretation when using blood-based protein measures as proxies for local central nervous system changes.

Some limitations in the current study need to be considered. First, despite a relatively large sample size, the number of individual proteins studied still limits power beyond overall correlations patterns. Second, the used inflammation panel (OLINK) also includes a mixture of well-established biomarkers and more exploratory markers but is still limited in terms of coverage for more complex immune signaling. Combined with the lack of experiments, this limits more detailed biological interpretations of underlying mechanisms for our associations. Further, some of the analytes in this panel, such as STAMBP, are sensitive to preanalytical handling, especially if measured in plasma [20–22]. However, in the current within-subjects analyses, STAMBP displayed no major deviation from the overall correlation pattern within and across conditions. Finally, while we attempted to control for confounders identified in our dataset and the existing literature, through statistical adjustment and sensitivity analyses in drug-naïve patients, the possibility of residual confounding cannot be entirely excluded.

## Conclusion

This study demonstrates distinct ex vivo association patterns for C4A and C4B in humans, revealing FEP-specific rewiring of C4A interactions with classical complement (C1Q) and inflammation-related proteins, in contrast to largely preserved C4B correlation patterns. The C4A–C1Q “uncoupling”, alongside prior experimental evidence, raises the possibility of complement dependent (C4A) but C1Q-independent synaptic tagging under certain pathophysiological conditions, possibly indicating a role for the lectin pathway. Concurrently, a selective positive shift in associations between C4A in FEP and proteins that are indirectly or directly inducible by IL-1β- (STAMBP, CCL20, CD40, 4E-BP1, and CCL25) aligns with experimental evidence of IL-1β-driven, selective neuronal C4A expression, potentially amplifying genetically driven increases in C4A protein levels in early schizophrenia.

## Supplementary information

Supplementary materials

## Data Availability

In line with institutional guidelines and Swedish legislation, raw data containing sensitive or personally identifiable information cannot be publicly shared due to data protection laws and policies. However, access to such data may be granted upon request to the corresponding authors, subject to applicable legal and ethical approvals on a case-by-case basis.
