# Supplementary material for: Divergence of C4A and C4B in first-episode psychosis: Insights from CSF and plasma immune profiling
Source: Transl Psychiatry. 2026 Apr 18;16:236. doi: 10.1038/s41398-026-04037-y (PMC13096202; doi:10.1038/s41398-026-04037-y)

**Supplementary materials**

**Divergence of C4A and C4B in first-episode psychosis: Insights from CSF and plasma immune profiling**

**Authors: Shokouh Arjmand^1,2*^, Mridul Chaudhary^1^, Samudyata^1^, Fredrik Piehl^3^, Aurimantas Pelanis^4^, Göran Engberg^1,5^, Simon Cervenka^6,7^, Mikael Landén ^8,9^, Sophie Erhardt^1^, Carl M. Sellgren^1,6*^**

^1^ *Department of Physiology and Pharmacology, Karolinska Institutet, Stockholm, Sweden*

*^2^ Translational Neuropsychiatry Unit, Department of Clinical Medicine, Aarhus University, Aarhus, Denmark*

^3^ *Neuroimmunology Unit, Department of Clinical Neuroscience, Karolinska Institutet, and Neuro Division, Karolinska University Hospital, Stockholm, Sweden*

^4^ *Department of Anesthesiology, Sahlgrenska University Hospital, Gothenburg, Sweden*

*^5^ Institute of Sport Science and Innovations, Lithuanian Sports University, Kaunas, Lithuania*

*^6^ Centre for Psychiatry Research, Department of Clinical Neuroscience, Karolinska Institutet & Stockholm Health Care Services, Region Stockholm, Sweden*

*^7^ Department of Medical Sciences, Psychosis Research and Preventive Psychiatry, Uppsala University, Uppsala, Sweden*

*^8^ Institute of Neuroscience and Physiology, University of Gothenburg, Gothenburg, Sweden*

*^9^ Department of Medical Epidemiology and Biostatistics, Karolinska Institutet, Stockholm, Sweden*

*Correspondence to: Shokouh Arjmand ([shokouh.arjmand@ki.se](mailto:shokouh.arjmand@ki.se)) & Carl M. Sellgren ([carl.sellgren@ki.se](mailto:carl.sellgren@ki.se))

C3 Fysiologi och farmakologi, C3 FyFa Cellulär modellering av psykiatriska sjukdomar, 171 77 Stockholm

| Table S1: *Demographic and clinical characteristics of participants, stratified by the cohorts, GRIP and KaSP.* | | | | | | | | | | | |
| --- | --- | --- | --- | --- | --- | --- | --- | --- | --- | --- | --- |
|  | | | | | | 95% Confidence Interval Mean | | | |  | |
|  | |  | | Mean | | Lower | | Upper | | Std. Deviation | |
| Sex (F/M) |  | GRIP |  | 97 (36/61) |  | - |  | - |  | - |  |
|  |  | KaSP |  | 106 (47/ 59) |  | - |  | -- |  | - |  |
| Age |  | GRIP |  | 40.89 |  | 38.16 |  | 43.62 |  | 13.54 |  |
|  |  | KaSP |  | 28.60 |  | 27.19 |  | 30.02 |  | 7.34 |  |
| BMI |  | GRIP |  | 25.65 |  | 24.92 |  | 26.39 |  | 3.62 |  |
|  |  | KaSP |  | 23.79 |  | 22.99 |  | 24.58 |  | 4.06 |  |
| Nicotine use |  | GRIP |  | 23 |  | - |  | - |  | - |  |
|  |  | KaSP |  | 21 |  | - |  | - |  | - |  |
| PANSS Positive |  | GRIP |  | 13.91 |  | 11.45 |  | 16.38 |  | 5.70 |  |
|  |  | KaSP |  | 18.88 |  | 17.47 |  | 20.29 |  | 5.77 |  |
| PANSS Negative |  | GRIP |  | 15.49 |  | 12.90 |  | 18.06 |  | 5.97 |  |
|  |  | KaSP |  | 16.85 |  | 15.19 |  | 18.51 |  | 6.80 |  |
| PANSS General |  | GRIP |  | 32.13 |  | 27.98 |  | 36.28 |  | 9.60 |  |
|  |  | KaSP |  | 37.31 |  | 34.75 |  | 39.88 |  | 10.51 |  |
| PANSS Total |  | GRIP |  | 61.52 |  | 54.62 |  | 68.43 |  | 15.96 |  |
|  |  | KaSP |  | 73.04 |  | 68.37 |  | 77.72 |  | 19.15 |  |
| GAF-S |  | GRIP |  | 84.96 |  | 82.92 |  | 87.00 |  | 7.26 |  |
|  |  | KaSP |  | 34.09 |  | 31.44 |  | 36.74 |  | 10.95 |  |
| GAF-F |  | GRIP |  | 85.31 |  | 83.32 |  | 87.31 |  | 7.09 |  |
|  |  | KaSP |  | 42.31 |  | 39.02 |  | 45.60 |  | 13.58 |  |
| CGI |  | GRIP |  | 4.60 |  | 2.72 |  | 6.48 |  | 1.52 |  |
|  |  | KaSP |  | 4.52 |  | 4.23 |  | 4.81 |  | 1.18 |  |
| DUP (months) |  | GRIP |  | NAv |  |  |  |  |  |  |  |
|  |  | KaSP |  | 11.26 |  | 6.56 |  | 15.95 |  | 17.85 |  |
| Antipsychotics use |  | GRIP |  | 38 |  | - |  | - |  | - |  |
|  |  | KaSP |  | 39 |  | - |  | - |  | - |  |
|  | | | | | | | | | | | |
| Note.  Excluded 2 rows from the analysis that correspond to the missing values of the split-by variable Cohort | | | | | | | | | | | |

| Table S2: *Missing values in the study* | | | | | | | | | | | | | |
| --- | --- | --- | --- | --- | --- | --- | --- | --- | --- | --- | --- | --- | --- |
|  | | C4A | | | | C4B | | | | C1QA | | | |
|  | | HCs | | FEPs | | HCs | | FEPs | | HCs | | FEPs | |
| Valid |  | 83 |  | 110 |  | 88 |  | 111 |  | 90 |  | 113 |  |
| Missing |  | 7 |  | 3 |  | 2 |  | 2 |  | 0 |  | 0 |  |
|  | | | | | | | | | | | | | |


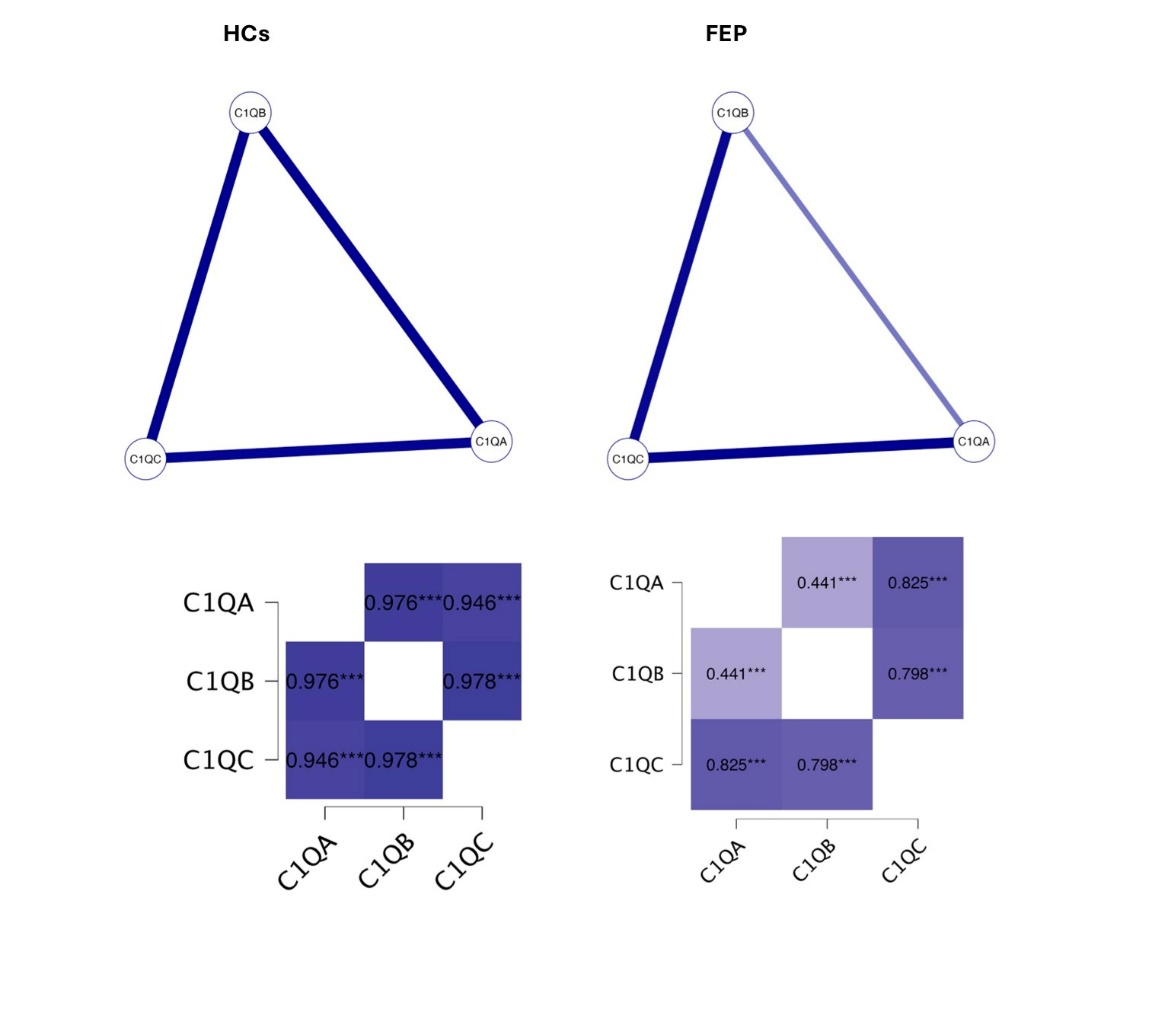


**Fig S1** | Network analyses and correlation matrices of the associations between CSF protein levels of C1QA, C1QB, and C1QC in healthy controls (HCs) and first-episode psychosis (FEP) patients adjusted for sex, age, and BMI.

In HCs, C1Q subtypes exhibit strong inter-correlations. Although the correlations remain statistically significant in FEP patients, their strength is comparatively reduced (large for C1QA-C1QC as well as C1QB-C1QC, and moderate for C1QA-C1QB).

**Fig S2|** **Correlations between nicotine use and the complement proteins.**

Reported p values for C1QA-NU (p = 0.948), C4A-NU (p = 0.379) and C4B-NU (p = 0.357).


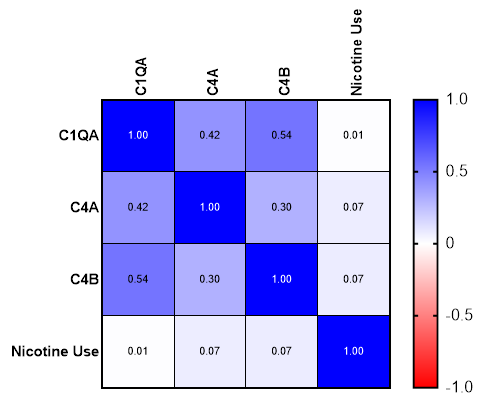

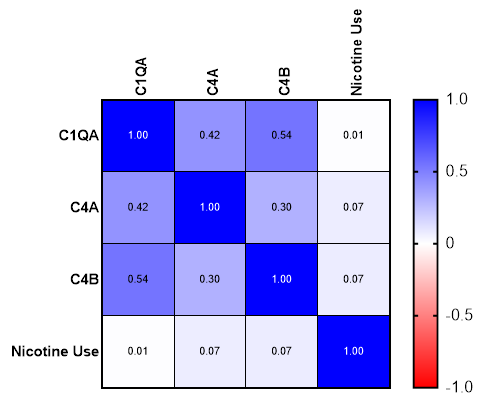

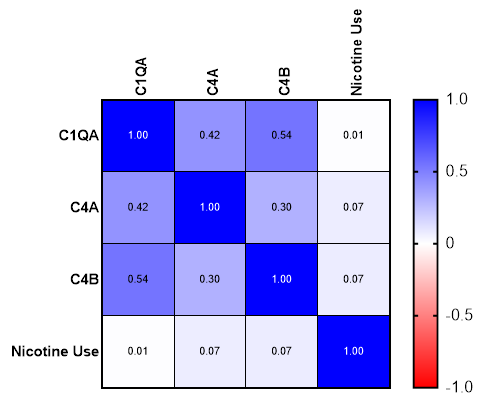


**Fig S3|** **Sensitivity analyses of antipsychotic medication use, with and without including antipsychotic exposure as a covariate (1), as well as analyses restricted to antipsychotic-naïve patients (2).**

(1)


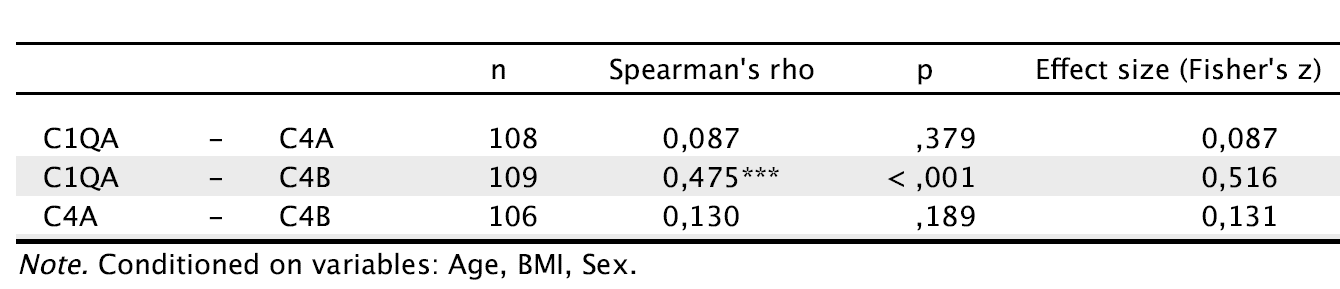


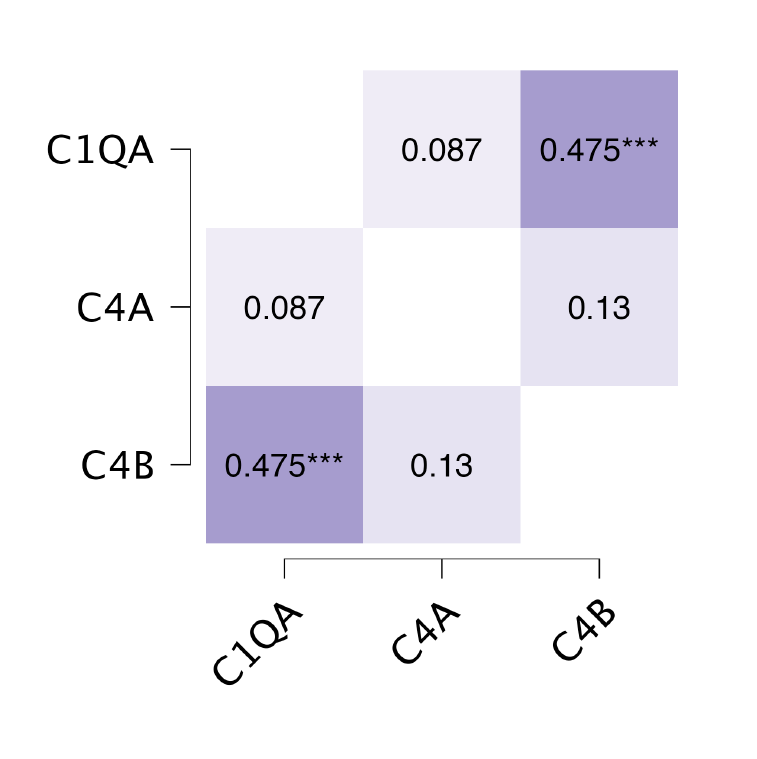


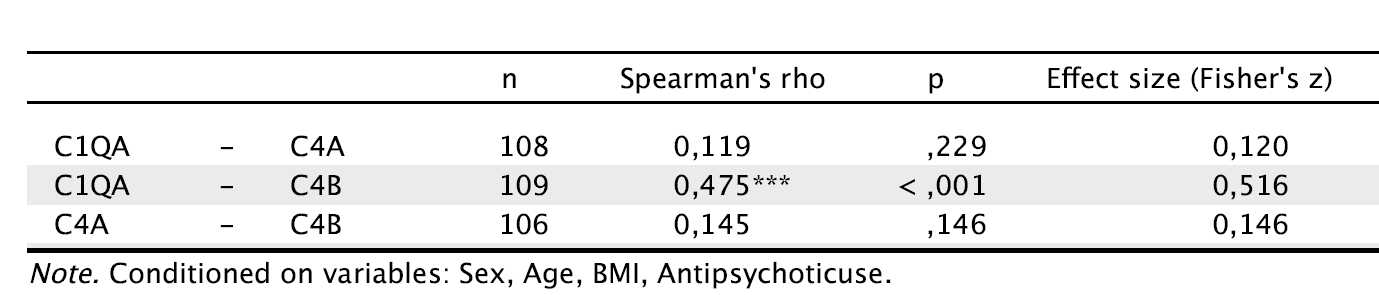


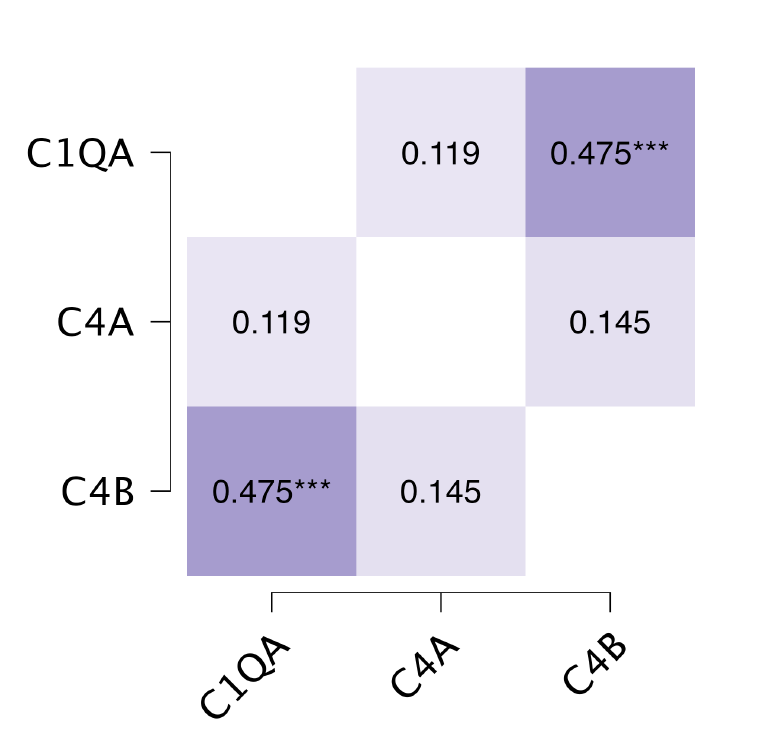


(2)


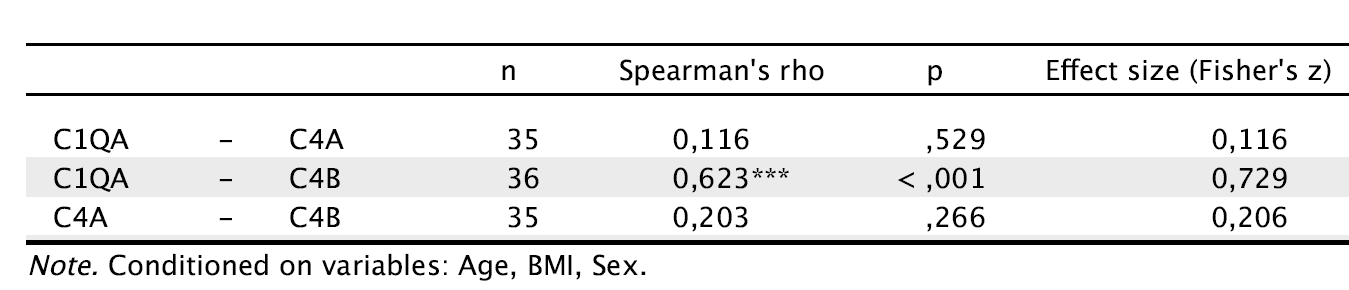

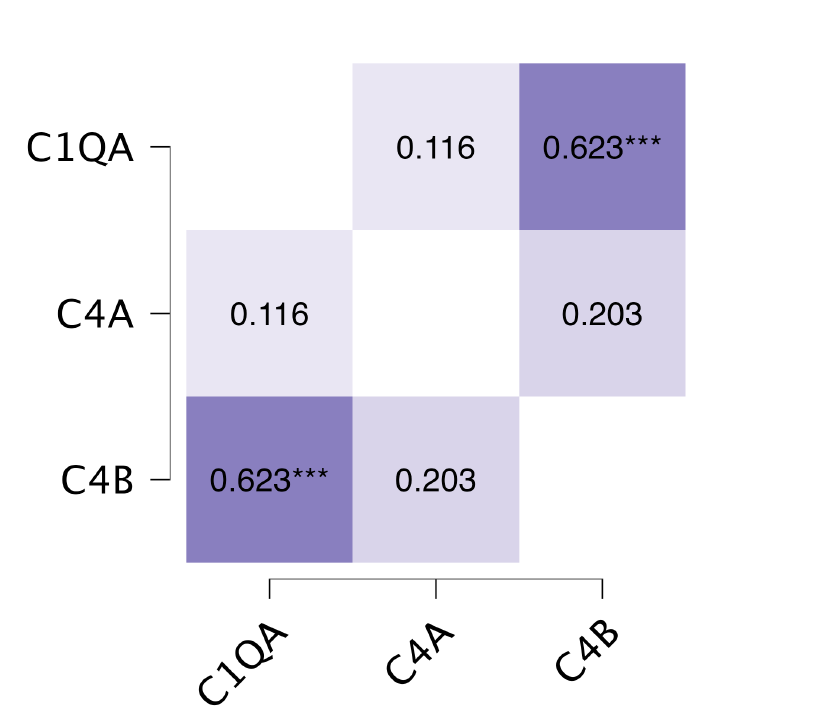


(1) We performed a sensitivity analysis to check for the possible effect of antipsychotic medications on the results. We controlled for the use of antipsychotic medications, and it did not affect the interpretation of the results. (2) Restricting the analysis to only antipsychotic-naïve patients did not influence the pattern of correlations either.

**Fig S4** **|** **Correlations of CSF C4A and C4B protein levels with CSF inflammatory markers, adjusted for gene copy numbers (CNs) in healthy controls (HCs) and patients with first-episode psychosis (FEP)**

Heatmaps comparing the association of CSF levels of C4A and C4B and 48 inflammatory proteins, before and after adjusting for gene CN, in HCs and patients with FEP (A). Positive and negative correlations are represented by cool and warm colors, respectively, with the strength of the correlation indicated by the intensity of the color.

Statistically significant correlations are illustrated in red. Correlations that did not remain significant after FDR correction are indicated by triangles, while those that survived FDR correction are indicated by squares. Forest plots, illustrating the correlations between CSF OLINK inflammatory markers and CSF levels of complement factors C4B and C4A in HCs 4B and in patients with FEP (B) after adjustment for the corresponding gene CNs. A network analysis of the CSF proteins demonstrating a strengthened correlation after adjusting for C4A CNs in HCs (C).

In general, the correlation patterns remained similar both for C4A and C4B in HCs and in patients with FEP. The few proteins that displayed a directional shift in the correlations, when adjusted for C4B in HCs, had modest initial effect sizes (Fig S4B). For C4A correlations, we observed a tendency towards pronounced negative correlations in HCs, with a similar correlation pattern as in the non-adjusted correlations for patients with FEP (Fig S4B). Adjusting for C4A in HCs strengthened correlations with several CSF with C4A levels, including MCP-1, TGF-alpha, FGF-5, LIF-R, PD-L1, DNER, TWEAK, and CSF-1 (Fig S4C), although none remained significant after FDR correction. In contrast, in FEP patients, adjusting for either C4B or C4A CNs did not alter the overall correlation patterns (Fig S4B).


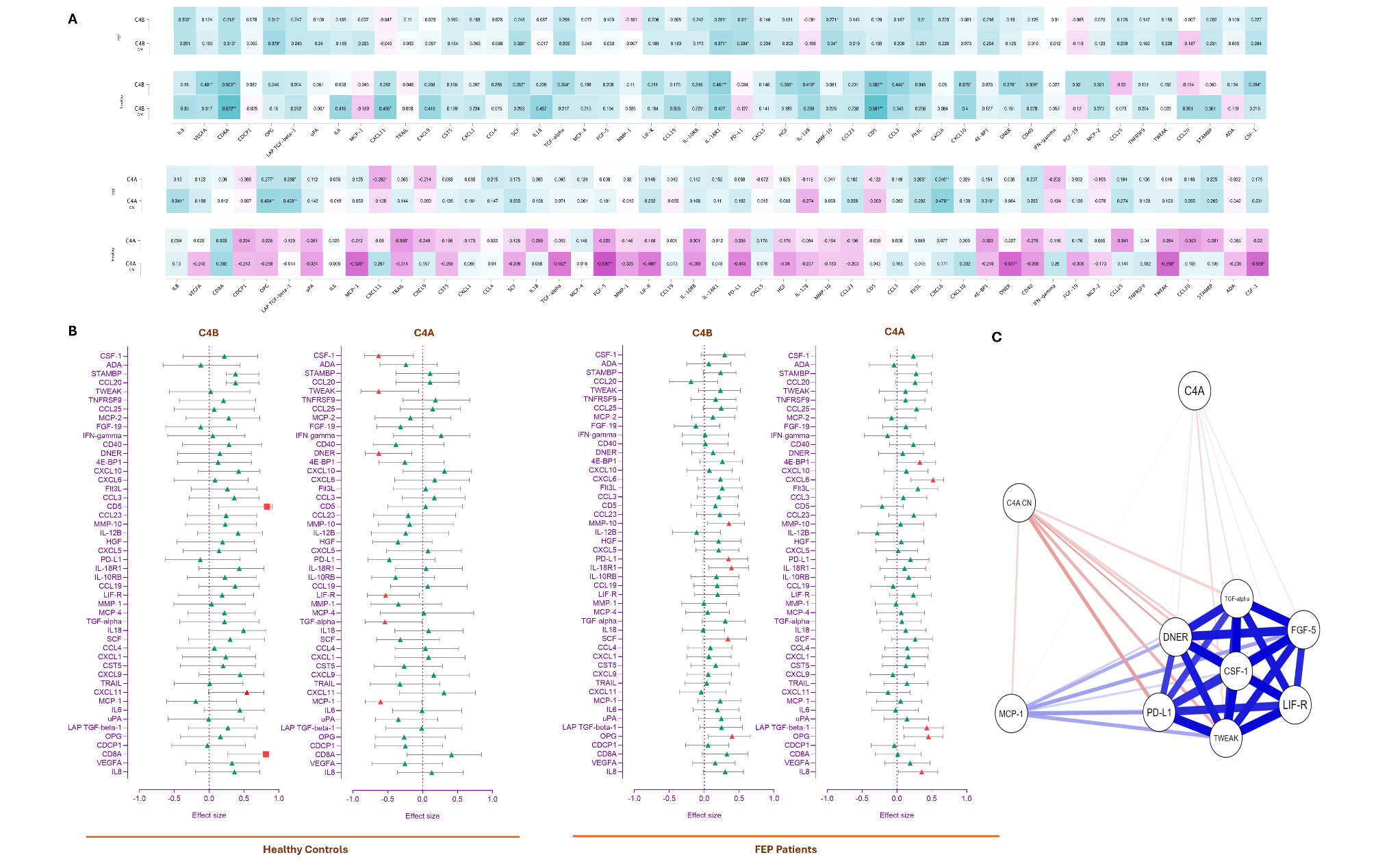


**Fig. S5 | Associations between CSF complement proteins (C4A, C4B, and C1QA) and plasma OLINK inflammatory markers in healthy controls (HCs) and patients with first-episode psychosis (FEP).**

Heatmaps displaying partial correlations between CSF levels of C4A, C4B, and C1QA and several Olink inflammatory proteins detected in plasma, adjusted for BMI, in HCs and FEP patients. Positive correlations are shown with cool colors, while negative correlations are indicated by warm colors, with the intensity of the color reflecting the strength of the correlation.


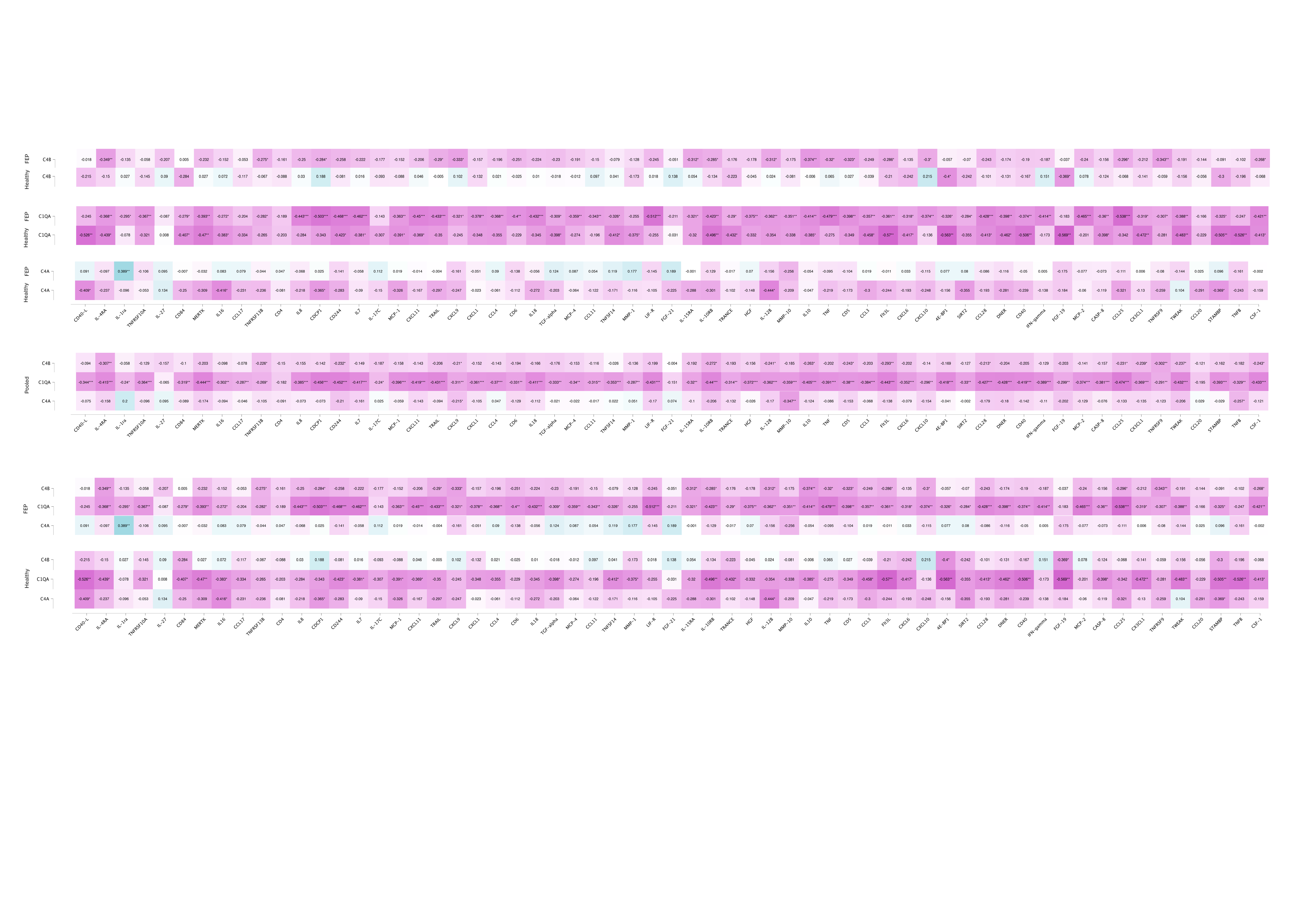

Supplement: Supplementary file 1 — Supplementary materials [file 41398_2026_4037_MOESM1_ESM.docx]
